# A meta-analysis of simulator sickness as a function of simulator fidelity

**DOI:** 10.1007/s00221-022-06485-6

**Published:** 2022-10-19

**Authors:** Ksander N. de Winkel, Tessa M. W. Talsma, Riender Happee

**Affiliations:** Department of Cognitive Robotics, Delft University of Technology, Mekelweg 2, Delft, 2628CD Zuid-Holland The Netherlands

**Keywords:** Driving, Simulator, Motion, Sickness, Fidelity, Kinetosis

## Abstract

Driving simulators are an increasingly important tool to develop vehicle functionalities and to study driver or passenger responses. A major hindrance to the use and validity of such studies is Simulator Sickness (SS). Several studies have suggested a positive relation between improvements in simulator fidelity and the likelihood of sickness. We hypothesized that this relation only holds true for static (fixed-base) simulators, and that increased fidelity in fact reduces simulator sickness in dynamic (moving-base) simulators. We performed a meta-analysis investigating the relation between sickness and fidelity in static and dynamic systems. A literature search yielded a total of 41 simulator studies that varied aspects of mechanical and/or visual fidelity and assessed SS for the same driving conditions and the same or equivalent participant groups. Evaluation of a model synthesizing the findings of these studies indicates that SS decreases with visual fidelity, and suggests that this effect may be negated for static simulators. The results of the modeling efforts thereby provide some support for the hypothesis that increased fidelity can reduce SS in dynamic simulators. Based on the evaluation of the literature we also note particular shortcomings and gaps in available research. Finally, we make recommendations for specific experiments that may fill these gaps and allow definitive conclusions on the role of simulator fidelity in SS.

## Introduction

Driving simulators are an increasingly important tool to develop (automated) vehicle functionalities, for driver training, entertainment, and to test human factors responses (Brems et al. 2015). The use of simulators has several advantages compared to real-road driving, including controllability, reproducibility, efficiency of data collection and safety (De Winter et al. 2012). A limitation of simulator studies is the occurrence of *Simulator Sickness* (SS^1^) (De Winter et al. 2012; Stanney et al. 1998), which is a particular form of motion sickness. To ensure the feasibility and validity of simulator studies, it is essential to understand the causes of motion sickness, and therefore SS.

The most prominent theory on the origin of motion sickness explains its occurrence as resulting from a conflict between actual and expected neural inputs of motion based on previous experience (Reason and Brand 1975). In light of this theory, sickness in a real vehicle, henceforth referred to as *Car Sickness* (CS), may occur when sensations of motion as generated by the visual, vestibular and somatic sensory systems, differ from what is expected based on previous experience. As intuitive examples, consider cases where visual and inertial sensations of motion mismatch; for instance when riding in a static (fixed-base) driving simulator, or similarly, when driving in a dynamic (moving-base) simulator with a limited motion envelope. Here, patterns of visual and vestibular afferent signals do not match patterns that have become familiar through experience.

SS, then, can be subdivided into *Simulated Car Sickness* (SCS), which is any motion sickness that results from simulator motion exactly as it would result from vehicle motion during actual driving, and *Simulator-Induced Sickness* (SIS), which results from false or missing motion cues. These can either be discrepancies between the actual simulator motion and expectations of typical vehicle motion based on previous experience, or discrepancies between motion cues provided by means of virtual imagery and inertial motion cues, again with previous experience as referent condition.

It may be argued that validity of driving simulator studies requires any SCS to closely approximate CS for any given driving scenario, whereas SIS should be minimized. It also stands to reason that these requirements would be met in the theoretical case of a motion simulator with a perfect *fidelity*, which exactly replicates real-world visual, inertial, tactile, and auditory referent stimuli within the simulator environment, and which induces a complete sense of immersion, referring to the state and extent of the mental involvement in a scenario (Agrewal et al. 2020).

Interestingly, previous studies on the relation between motion sickness and simulator fidelity have yielded some—when taken at face value—counterintuitive findings: notably, an absence of sickness in static motion simulators and a *positive* relation between fidelity and SS, suggesting that sickness increases for higher levels of fidelity (Miller and Goodson 1960; McGuinness et al. 1981; Kolasinski 1995; Jäger et al. 2014; Seay et al. 2001; Lin et al. 2002a; Stoffregen et al. 2000; Smart Jr. et al. 2002; De Winter et al. 2007, 2012; Lee 2004; Blissing and Bruzelius 2018; Ledegang et al. 2015). Such observations imply that low-fidelity simulators may be preferable. For example, Lee (2004) argued that the pursuit of higher levels of fidelity in simulators may be counterproductive because it can increase SS: “In fact, low-fidelity simulators or simulators that intentionally distort the driving experience may be more effective than those that strive for a veridical representation of the driving environment” (cited in: de Winter (2009)). Although this conclusion might apply in certain cases, such as studies on transfer-of-training (Liu et al. 2008), it is problematic for studies on CS, because minimization of overall SS also minimizes SCS. Moreover, the argument does not take into account that the referenced studies were performed on static simulators.

On the one hand, absence of symptoms in a static simulator with low-fidelity imagery (Miller and Goodson 1960) could be due to the stimuli failing to induce any sensations of self-motion (De Winkel et al. 2017, 2018a), which is thought to be a requirement for motion sickness to develop (Nooij et al. 2017); but on the other hand, high fidelity virtual imagery that *does* induce compelling sensations of self-motion may actually increase the magnitude of the conflict between the visual and (absent or scaled) physical motion, resulting in increased SIS when low SCS would be expected (Seay et al. 2001; Lin et al. 2002a; van Emmerik et al. 2011; Kaufeld and Alexander 2019).

Therefore, the view that low-fidelity simulators may be preferable may have to be nuanced when considering studies on motion sickness. As an alternative take on the relation between fidelity and SS, we consider findings by Chang et al. (2020) on the relation between the fidelity of Virtual Reality (VR) systems and (visually induced) motion sickness. These authors report decreased discomfort for high fidelity visual stimulation *if* the VR provided multisensory stimulation, for instance in the form of additional vestibular, auditory or proprioceptive cues. However, when only visual information was provided, increased visual fidelity increased sickness.

Similarly, we hypothesize that (1) increasing visual fidelity in a static simulator increases SS, whereas (2) increasing visual fidelity in a dynamic simulator will reduce SS.

We evaluate these hypotheses by performing a meta-analysis of studies that compare motion sickness for a given scenario between at least two experimental conditions wherein some technological aspect of fidelity is varied, and that were performed either on a static or dynamic simulator. To test the hypotheses, we evaluate whether the direction of any relation between fidelity and motion sickness is different between static and dynamic simulators. Specifically, we expect this relation to be positive in the former case, and negative in the latter.

## Methods

### Literature search

The literature was searched using Scopus and, given its particular relevance to the present topic, the proceedings of the Driving Simulation Conference.

For the Scopus search, the search terms were derived from the research questions:

The terms “simulator sickness”, “motion sickness”, “cyber sickness”, “car-sickness”, “sea-sickness”, “air-sickness”, and “kinetosis” were used to represent the dependent variable.

As independent variable(s) we consider the general term “fidelity”, and key features of different aspects of *visual-audio fidelity* and *motion fidelity* as defined by Liu et al. (2008) (see also the section on “Fidelity rating”).

For visual-audio fidelity, we included terms for screen size: “Field-of-View” (FoV); for modality we included: “monitor”, “screen”, “projection”/“projection system”, “HMD”; for the availability of stereoscopic cues: “2D”, “3D”, “monoscopic”, “stereoscopic”; for scene content: “realism”, “graphics”, “scenario”; and finally, we included “resolution”.

Motion fidelity is the correspondence between mechanical/inertial accelerations experienced in the simulator and in the real vehicle for a given trajectory. We discriminate on a high level by comparing static to dynamic simulators, but for motion simulators we also consider the simulator “degrees-of-freedom” (DoF) and “motion cueing” algorithm, because these relate to the hardware and software capabilities of a motion base.

Terms for the dependent and independent variables were bracketed, separated by the term“OR”; with an“AND” between the bracketed strings for the dependent variables and the independent variables, such that the search string was:

((“motion sickness” OR “simulator sickness” OR “cyber sickness” OR “cybersickness” OR “car sickness” OR “air sickness” OR “seasickness” OR “kinetosis”) AND (“fidelity” OR “Field-of-View” OR “Field of View” OR “monitor” OR “screen” OR “projection” OR “projection system” OR “HMD” OR “2D” OR “3D” OR “monoscopic” OR “stereoscopic” OR “realism” OR “graphics” OR “scenario” OR “resolution” OR “degrees of freedom” OR “degrees-of-freedom” OR “motion cueing”))

The search was last performed June 27, 2022, and yielded 1282 results. To narrow down these results, the term “simulator” was included, resulting in 558 results; We also evaluated a search including the additional term “driving”, yielding 151 results.

In addition to the Scopus search, we searched the proceedings of the Driving Simulation Conference (DSC) using the search terms “sickness” and “kinetosis”.

The titles produced by the searches were screened, and for titles that provided an approximate match with the criteria the abstracts were read. The primary eligibility criteria for inclusion were that studies were:

1. written in English,
2. at most 20 years old,
3. peer-reviewed,
4. empirical work using human participants,
5. in a paradigm that involved vehicle motion,
6. reporting sickness metrics,
7. comparing at least two experimental conditions with varying aspects of fidelity for the same scenario.

Studies also had to provide sufficient detail on the methodology for the dimensions of visual and motion fidelity under study to be characterized or quantified, and SS data had to be sufficiently detailed to derive at least one overall sickness metric with a known scale per experimental condition. The latter requirements were judged after reading of each study.

For studies found suitable, data were extracted either from relevant tables or graphs, or was provided by the authors upon our request. Where authors were unresponsive, studies were excluded from the analyses. In total, suitable data was obtained from 41 studies.

All studies that were screened are listed in the spreadsheet document that is available as supplementary material, along with any data obtained and used for subsequent analyses.

### Fidelity rating

A number of fidelity rating systems have been proposed previously. As an example, we consider the system proposed by Wynne et al. (2019). In the original system, the fidelity of a simulator is rated using three fidelity measures:

1. the nature (single vs. multiple monitors, or projectors) and FoV (in bins, $<180^\circ$, $180^\circ -270^\circ$, $>270^\circ$) of the visualization system,
2. the absence or presence, and Degrees-of-Freedom (DoF) of a motion base, and
3. the physical realism of a simulator (ranging between sitting at a desk to being seated in a vehicular cabin),

with each measure being given 1–5 points. Because the ranges are equal, each measure has equal weight to the total rating, which can take on values between 3 and 15. The rating system allows placement of a simulator along a fidelity continuum. On the lower end would be the lowest fidelity simulator, existing of for example a single computer screen and a seat. At the opposite end would be high-fidelity simulators, consisting of a motion base, vehicle cabin and large FoV projection screens surrounding the cabin.

There are some limitations to this (and similar) rating system(s). The primary issue is that these systems necessarily rely on a number of more-or-less arbitrary choices; given the definition of simulation fidelity, ratings of particular modalities should capture the extent to which some technological aspect accurately reproduces its real-world counterpart. Whereas the FoV and the number of screens, or the presence or absence of a motion base and its DoF arguably *contribute* to visual fidelity, it is not clear how variations of individual characteristics affect aspects of fidelity; how continua may be binned; or how different dimensions should be weighted to reproduce the experience of fidelity. To deal with these issues, we first separately consider *Visual fidelity*; *Mechanical fidelity*, referring to the realism of mechanical/inertial motion; and a number of peripheral factors affecting either fidelity or SS (Liu et al. 2008).

#### Visual fidelity

In addition to monitors and projection systems, visualizations may also be produced by an out-the-window view, or by using head mounted displays. Although an out-the-window view by definition provides the highest visual fidelity achievable, the ordering of the other systems in terms of their contribution to an experience of fidelity is not known, and this ordering may also be affected by specifics of the particular system. For example, on the one hand, Head-Mounted Displays (HMDs) might offer the highest degree of immersion out of the existing visualization technologies, but on the other hand, HMDs introduce detrimental latencies (e.g., related to head tracking) and issues related to the lenses, which do not apply in the same way to other technologies.

The binning of the FoV proposed by Wynne et al. (2019) is arbitrary. An alternative method to bin FoV would be to apply a division of the optical array into central ($<60^\circ$, (Strasburger et al. 2011)) and peripheral vision ($60^\circ -180^\circ$), and to add a ‘full FoV’ category for systems with even larger FoV that allow looking around. This division is likely relevant to fidelity, because sensations of self-motion are induced primarily through peripheral vision (Pretto et al. 2009; De Winkel et al. 2018b). Instead, it is also plausible that fidelity increases as a function of the size of the FoV. We will assess how observations of SS vary depending on the classification, and whether a correlation exists between horizontal FoV and SS.

Other factors that may affect visual fidelity reflect the quality of the equipment in relation to human vision. Specifically, whereas humans have 3D vision thanks to biocular stereoscopy, typical monitors and projection systems render only a single visual virtual environment visible to both eyes, which is a 2D stimulus; furthermore, the maximum visual acuity of the human eye is approximately 128 pixels per degree (PPD, Deering (1998)). Individual pixels may be visible for systems with a lower PPD. It is likely that fidelity increases with the number of PPD. Finally, the frame rate is a factor that likely affects fidelity. Humans start perceiving continuous motion from sequentially presented images at about 24 Hz, but appear able to detect fluctuations up to 500 Hz (Davis et al. 2015). Similar to the role of FoV and PPD, it is likely that more Frames-Per-Second (FPS) produce higher fidelity. We will contrast observations on motion sickness for stereoscopic and non-stereoscopic visualizations, and assess whether there is a relation between SS and FoV, PPD, and FPS. For out-the-window viewing conditions, we adopted maxima of 128 PPD and 500 FPS (Deering 1998; Davis et al. 2015). These values were included to provide a reference condition for perfect fidelity.

#### Mechanical fidelity

The quality of simulated mechanical motion ultimately may be expressed by the extent to which presented mechanical accelerations match a trajectory of target vehicle accelerations. However, studies typically do not report sufficient detail on the cueing that was used nor on the generated accelerations to estimate such correspondence. Moreover, as there is an infinite number of ways for a mechanical motion trajectory to differ from a target trajectory, it is not apparent how such correspondence should be quantified. Therefore, we will contrast *static* and *dynamic* simulators, and evaluate whether there is any apparent relation between SS and the DoF of a simulator motion base.

#### Peripheral factors

We will distinguish simulators according to their physical layout using a subset of the categories used by Wynne et al. (2019), namely desktop-setups, mockups, (half-)cabins or real vehicles. We have encountered a number of conditions that cannot be readily classified as either of these layouts; for example, a study with participants lying supine while viewing a large projection screen via a mirror (Emoto et al. 2008). Such uncommon conditions will be classified as ‘other’. We will also consider a number of covariates that may account for variance in the SS data. We will consider whether participants were active (drivers) or passive (passengers), as experimental work where participants were exposed to rotational stimuli has shown that participants who had no control over the stimuli were more likely to experience motion sickness than participants who did have control (Rolnick and Lubow 1991); we will take into account the duration of provocative stimulation; and the average age of the sample. Although it has been reported that there are sex differences in motion sickness susceptibility (see e.g., Lackner (2014)), it was not possible to assess such effects in this analysis because studies tend to only report the ratio of male/female participants, but rarely differentiate their findings based on participant sex, except for single cases where this is a specific research question (Garcia et al. 2010).

### Sickness scoring

Various methods exist to quantify motion sickness. These can be subdivided into physiological and subjective methods. Although physiological methods can be considered objective, there are issues with their reliability and specificity (De Winkel et al. 2022). Consequently, subjective methods, in the form of a variety of rating scales, are as of yet the preferred method to quantify motion sickness.

The majority of studies included in this analysis (31/41) have used the Simulator Sickness Questionnaire (SSQ, Kennedy et al. (1993)) to quantify motion sickness symptoms. This questionnaire divides motion sickness symptoms into three factors, namely *Nausea*, *Oculomotor* (relating to issues with vision) and *Disorientation*. The scale features 16 Likert-scale items, each with integer scores ranging between 0 and 3. Scores can be calculated for the factors by summing the scores on subsets of seven items that load on these factors, and weighting the results by coefficients of 9.54, 7.58, or 13.92, respectively. A total score can be calculated by adding the *unweighted* factor scores, and multiplying by 3.74. Thus, the total score can range between 0 and 235.62 (i.e., $3 \times (3\times 7) \times 3.74$). Other studies instead used either the Motion Sickness Assessment Questionnaire (MSAQ, Gianaros et al. (2001); 1/41), which has a range of 16–144 on the total score that is then converted to a percentage; a Motion Sickness Questionnaire, which ranges between 0 and 78 (MSQ, Frank et al. (1983); 1/41); the Fast Motion Sickness scale, ranging between 0 and 20 (FMS, Keshavarz and Hecht (2011); 3/41), or some variation of a magnitude estimation (ME) scale (e.g., (Hartfiel and Stark 2019; Aykent et al. 2014); 5/41). Because two studies used both the SSQ and the FMS (Sawada et al. 2020; Keshavarz and Hecht 2012), there are 43 methods for 41 studies. What these scales have in common is that they feature absolute minima and maxima. To be able to combine data from different metrics in a single analysis, we thus assume that each scale can be interpreted as a reflection of a latent sickness variable that ranges between an absolute minimum and a maximum that corresponds to ’frank sickness’, which is a theoretical culmination of motion sickness in vomiting and a maximum misery (De Winkel et al. 2022). We normalize the scores such that they range between 0 and 1 by using the extremes of each scale. In contrast to the questionnaire based methods (SSQ, MSAQ, MSQ), the FMS and other ME methods require participants to summarize their experience of motion sickness in a single number. Data from the two studies that used both SSQ and FMS indicated that FMS scores were higher than SSQ scores ($\text {SSQ}_{\text {normed}} = 0.347 \text {FMS}_{\text {normed}} + 0.047$). This suggests that the distribution of scores may differ between questionnaire and ME-based methods, which could complicate converting scores to a common scale. We evaluated whether and how the findings of our analyses varied, depending on which of two methods to deal with this was used. First, we chose to use only the SSQ scores for the studies that measured both SSQ and FMS. Second, We evaluated the effect of applying the above formula to convert FMS and ME scores to the range of SSQ scores. The conclusions that could be drawn based on the outcome of the analyses were similar in size and direction, but in some cases did not reach statistical significance when using scaled ME responses. As the method appears more conservative, we present the findings for analyses using the second method in the main text, but note where using analyses using unscaled data yield different conclusions.

Sickness scores were retrieved either from mean and standard deviation (SD) or standard error of the mean (SEM) values reported in text or tables, or by reading out values from figures; using a vector drawing program to derive precise measurements.

From those studies that used the SSQ and that reported both the mean and standard deviation (SD; or instead the standard error of the mean, $\text {SEM}=\text {SD} / \sqrt{n}$), it was observed that there was a very strong correlation between the mean and SD ($\rho =0.880, p=2.141*10^{-37}$). A linear regression yielded the following relation: $\ln ({\text {SD}_\text {SS}})=-0.630+0.752\ln ({\text {SSQ}})$. Using this relation, we imputed the SD for studies where it was not reported and where it could also not be retrieved from figures (Park et al. 2005; Hohorst et al. 2019; Kim and Park 2020; Boustila et al. 2017). These imputed values were then used in the meta-regression (see: “Data analysis”). It may be worth noting that the SD tended to be approximately equal to the means.

Finally, while collecting the sickness scores, we noted apparent mistakes in the calculation of sickness metrics in several studies (Gemonet et al. 2021; Colombet et al. 2016; Jung et al. 2021; Zhang and Wang 2020; Ujike and Watanabe 2011; Garcia et al. 2010; Monteiro et al. 2020). A recurring mistake in the calculation of total SSQ scores was that *weighted* subscale scores were summed and then once again weighted with the coefficient for the total score, resulting in total scores beyond the range of the scale. In cases where the cause of mistake was apparent, it was corrected and data were included in the analyses. Studies where the mistake could not be resolved were excluded. Notes on applied corrections can be found in the supplementary spreadsheet document that contains the search results and extracted data.

### Data analysis

The data analysis is divided in two main parts. In the first part, we perform an exploratory analysis to evaluate relations between SS and individual aspects of fidelity. This analysis is aimed at corroborating consensuses on the effects of these variables by themselves. We fit mixed effects linear models with SS as dependent variable, each of the aspects of fidelity listed previously as independent variables in the form of fixed factors, and include a random intercept for each study and a random slope for the particular factor to account for variation in study designs. We then perform an ANOVA on the fixed factors to evaluate whether there are differences between the levels of the independent variables; similarly, in case of continuous predictors we evaluate whether there is a non-zero linear relation between $\ln ({\text {SS}})$ and the variable.

The exploratory part can be considered an evaluation of SS by contrasting the levels of individual factors. Due to covariation of different factors, there is a risk of confounding effects in this type of analysis. In the second part of the data analysis, we deal with this by performing a multivariate meta-regression, tailored to address our hypotheses. Whereas a *narrative* review can be interpreted as a synthesis of conclusions from other studies based on *p*-values, a meta-regression can be interpreted as a *quantitative* review that is a synthesis of the data of other studies (Borenstein et al. 2021). We start this analysis by assessing whether sufficient observations are available to test for interaction effects between the individual aspects as predictors of SS. We then perform mixed-effect regressions that include as *fixed effects* those aspects of fidelity for which sufficient observations are available, and we include *random effects* that account for variability of SS on the level of the study due to differences between studies that are not directly accounted for, such as susceptibility of participants in the study sample, the choice of scenario, or the intensity of a stimulation.

In meta-regression, observations are typically weighted by measures that reflect their relative importance or reliability Borenstein et al. (2021); Schmidt and Hunter (2014). Weighting schemes may be based on observations’ respective precisions, such as the inverse of the standard error of the mean ($\text {SEM}=\text {SD} / \sqrt{n}$). However, as noted previously, it was observed that there was a strong positive correlation between these metrics. Consequently, weighting by the inverse of this uncertainty would have the effect of attributing less weight to studies that provoke more sickness. As this is undesirable, we evaluate the effect of weighting by sample size instead (Schmidt and Hunter 2014).

To aid in the understanding of the general methodology, imagine a three-dimensional space, featuring an X-axis that represents ‘visual motion quality’, a Y-axis which represents ‘mechanical motion quality’, and a Z-axis that represents SS. As per the requirements for inclusion in the analysis, an individual hypothetical study will have assessed SS for a given scenario, while having varied some aspect of visual motion quality, mechanical motion quality, or both. Regardless of the specific experimental manipulations, the observed SS scores can be marked in this space, and along any dimension that was varied an effect can be estimated. The effect would be represented by a line if only visual or mechanical motion was varied, or a surface if both visual and mechanical motion were varied. We can then populate this space with the lines or surfaces from all the studies that meet the inclusion requirements. If other variables, such as the choice of scenario, were also identical between studies, then a single surface could be fitted through the cloud of effects, and conclusions on the role of fidelity could be drawn directly. However, due to such nuisance factors, there is additional variation in the location of the line or surface along the SS axis for each study, apart from the visual and mechanical motion quality dimensions. In the mixed-effect model, the dimensions that represent different aspects of simulator hardware are included as *fixed effects*, and the individual offsets due to nuisance factors are included as *random effects*. Hence, the modeling can be thought of as fitting a common surface through a multidimensional cloud of effects, while taking into account that the location of the surface along the vertical axis may vary between studies.

All regressions are performed using the natural logarithm of the SS scores, $\text {ln(SS)}$, as regressands. This transformation is performed to meet the assumption in ANOVA that residuals are approximately normal distributed. For figures, the non-transformed data will be shown, which allows for easier interpretation. Note that due to these transformations, the fits shown in the figures are not linear, and errorbars are not necessarily symmetrical around the means.

In addition to the meta-regression, we further analyse observations made in the exploratory analysis that SS is worst for visualizations presented in stereoscopic 3D and for HMD. Specifically, we evaluate whether there is evidence that stereoscopic visualizations and HMDs have additive effects, or whether these observations reflect confounding due to most 3D visualizations being generated using HMDs.

## Results

In the following, we summarize the findings of our literature search and meta-analysis. In the Exploratory analysis section, we report the number of observations per level of each aspect of fidelity, as described in the Fidelity rating section of the Method. Here we also evaluate how SS relates to these aspects in isolation of other aspects. In the second part of the results section, we report how many observations there are per combination of levels of each fidelity aspect, and we assess the granularity that may be achieved in a meta-regression of SS. Based on these findings, we implement a mixed-effect model and assess whether this allows validation of the hypotheses.

**Fig. 1:**
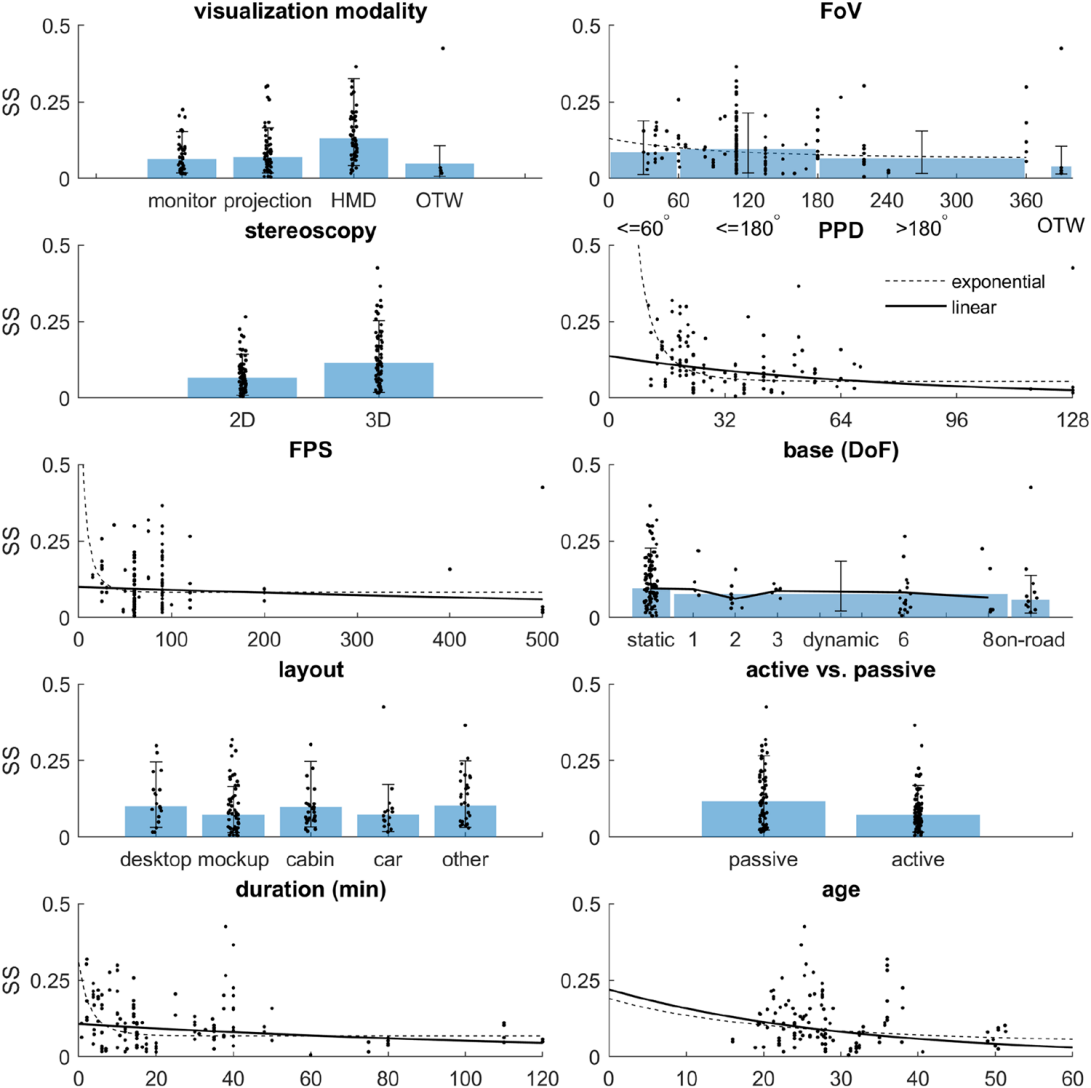
Comparison of marginal mean SS scores ($\pm \text {SD}$) between varying levels of different aspects of visual fidelity (modality, FoV, stereoscopy, PPD and FPS), mechanical fidelity (base), and peripheral factors (layout, active vs. passive driving, exposure duration and sample average age). Univariate ANOVAs performed for individual predictor variables indicate that there are differences in SS depending on: the visualization modality, with HMDs producing more sickness than other modalities; and whether or not visualizations are presented in stereoscopic 3D. Fitting a linear model, SS was also found to decrease with PPD. However, note how the relation between some continuous predictors (notably PPD) appears to decay exponentially rather than linearly. For FoV and PPD, an exponential decay model indeed provided a significantly better fit (dashed lines in respective panels). The significant effects for HMD and stereoscopic vs. non-stereoscopic visualization suggest that there may be a problem of multicollinearity, with most observations on stereoscopic 3D obtained using HMDs. This is evaluated in a separate analysis. Except for FoV, a small amount of jitter was added to the x-position of data points for the categorical variables in order to improve visibility. For FoV and DoF, the width of the bars was set to approximately match the size of the bins, with some white space between the bars to enhance the different groups

### Exploratory analysis

A total of 41 studies was retained for the analyses. From these studies, observations on SS were obtained for 148 experimental conditions, differing with respect to one or more aspects of fidelity. A visual summary of the relation between the levels of each of these aspects of fidelity and observations on SS is provided in Fig. 1. Descriptive statistics for each of these aspects are provided in the following paragraphs.

#### Visual fidelity

*Visualization modality* We distinguish between four types of visualization modalities. Out of all studies, 20 used monitors, in 36 conditions (Zou et al. 2021; Gemonet et al. 2021; Almallah et al. 2021; Parduzi et al. 2020; Suwarno et al. 2019; Hohorst et al. 2019; Walch et al. 2017; Romano et al. 2016; Bridgeman et al. 2014; Park et al. 2005; Sekar et al. 2020; Parduzi et al. 2019; Ujike and Watanabe 2011; Garcia et al. 2010; Kim and Park 2020; Stelling et al. 2020; Somrak et al. 2019; Mittelstaedt et al. 2018; Häkkinen et al. 2006; Klüver et al. 2016); 16 studies used projection systems, in 54 conditions (Talsma et al. 2022; Jurisch et al. 2020; Benz et al. 2019; Weidner et al. 2017; Aykent et al. 2014; Park et al. 2005; Lin et al. 2002b; Colombet et al. 2016; Parduzi et al. 2019; Will et al. 2017; Schmieder et al. 2017; Keshavarz and Hecht 2012; Boustila et al. 2017; Emoto et al. 2008; Damveld et al. 2010; Klüver et al. 2016); 21 studies used HMDs, in 54 conditions (Zou et al. 2021; Sawada et al. 2020; Parduzi et al. 2020; Suwarno et al. 2019; Benz et al. 2019; Walch et al. 2017; Weidner et al. 2017; Colombet et al. 2016; Lucas et al. 2020; Hartfiel and Stark 2019; Eftekharifar et al. 2021; Jung et al. 2021; Kim et al. 2020; Kaufeld and Alexander 2019; Moss and Muth 2011; Kim and Park 2020; Stelling et al. 2020; Somrak et al. 2019; Mittelstaedt et al. 2018; Häkkinen et al. 2006; Kovácsová et al. 2020); and 4 used an out-the-window view, with a total of 4 observed conditions (Talsma et al. 2022; Zou et al. 2021; Gemonet et al. 2021; Klüver et al. 2016). ANOVA indicated that there are differences between the conditions ($F(3,144)=9.751, p<0.001$, see Fig. 1). HMDs appear to provoke more SS than the alternative modalities. The marginal mean SS scores for these conditions were 0.064, 0.070, 0.131, 0.049, respectively.

*Field-of-View* Following the consensus on the subdivision of the human FoV, we binned studies with visualizations that covered only (part of) the central FoV $<60^\circ$, yielding eight studies with observations in 19 conditions (Benz et al. 2019; Bridgeman et al. 2014; Keshavarz and Hecht 2012; Moss and Muth 2011; Ujike and Watanabe 2011; Somrak et al. 2019; Emoto et al. 2008; Häkkinen et al. 2006); both the central and peripheral FoV $60^\circ -180^\circ$, yielding 29 studies and 89 conditions (Zou et al. 2021; Almallah et al. 2021; Sawada et al. 2020; Parduzi et al. 2020; Suwarno et al. 2019; Hohorst et al. 2019; Walch et al. 2017; Weidner et al. 2017; Romano et al. 2016; Aykent et al. 2014; Park et al. 2005; Colombet et al. 2016; Lucas et al. 2020; Sekar et al. 2020; Parduzi et al. 2019; Hartfiel and Stark 2019; Eftekharifar et al. 2021; Jung et al. 2021; Kim et al. 2020; Kaufeld and Alexander 2019; Keshavarz and Hecht 2012; Kim and Park 2020; Stelling et al. 2020; Somrak et al. 2019; Mittelstaedt et al. 2018; Boustila et al. 2017; Emoto et al. 2008; Kovácsová et al. 2020; Klüver et al. 2016); full FoV ($>180^\circ$), 11 studies and 36 conditions (Talsma et al. 2022; Gemonet et al. 2021; Jurisch et al. 2020; Lin et al. 2002b; Colombet et al. 2016; Parduzi et al. 2019; Will et al. 2017; Schmieder et al. 2017; Garcia et al. 2010; Damveld et al. 2010; Klüver et al. 2016); and studies with an actual OTW view, for which there were four studies, and four conditions (Talsma et al. 2022; Zou et al. 2021; Gemonet et al. 2021; Klüver et al. 2016). Neither a groupwise comparison ($F(3,144)=1.852, p=0.140$), nor the fit of a nonlinear exponential decay model of the form $\ln ({\text {SS}})=a\ + b\ \exp (-c\ \text {FoV})$, showed significant effects of FoV. The marginal means for these conditions were: 0.087, 0.096, 0.066 and 0.040, respectively.

*Stereoscopy* Thirty-three studies used (biocular) non-stereoscopic visualizations in 79 experimental conditions (Talsma et al. 2022; Zou et al. 2021; Gemonet et al. 2021; Almallah et al. 2021; Jurisch et al. 2020; Parduzi et al. 2020; Suwarno et al. 2019; Benz et al. 2019; Hohorst et al. 2019; Walch et al. 2017; Weidner et al. 2017; Romano et al. 2016; Bridgeman et al. 2014; Aykent et al. 2014; Park et al. 2005; Sekar et al. 2020; Parduzi et al. 2019; Will et al. 2017; Schmieder et al. 2017; Eftekharifar et al. 2021; Keshavarz and Hecht 2012; Moss and Muth 2011; Ujike and Watanabe 2011; Garcia et al. 2010; Kim and Park 2020; Stelling et al. 2020; Somrak et al. 2019; Mittelstaedt et al. 2018; Boustila et al. 2017; Emoto et al. 2008; Häkkinen et al. 2006; Damveld et al. 2010; Klüver et al. 2016), with a mean sickness score of 0.067; 28 studies used stereoscopic 3D, in 69 experimental conditions (Talsma et al. 2022; Zou et al. 2021; Gemonet et al. 2021; Sawada et al. 2020; Parduzi et al. 2020; Suwarno et al. 2019; Benz et al. 2019; Walch et al. 2017; Weidner et al. 2017; Lin et al. 2002b; Colombet et al. 2016; Lucas et al. 2020; Hartfiel and Stark 2019; Schmieder et al. 2017; Eftekharifar et al. 2021; Jung et al. 2021; Kim et al. 2020; Kaufeld and Alexander 2019; Keshavarz and Hecht 2012; Ujike and Watanabe 2011; Kim and Park 2020; Stelling et al. 2020; Somrak et al. 2019; Mittelstaedt et al. 2018; Boustila et al. 2017; Häkkinen et al. 2006; Kovácsová et al. 2020; Klüver et al. 2016), with a mean sickness score of 0.115. ANOVA indicated that there are differences between the conditions ($F(1,146)=10.832, p=0.001$, see Fig. 1). Visualizations presented in stereoscopic 3D appear to provoke more SS than the non-stereoscopic visualizations.

*Pixels-Per-Degree* To assess the effect of image resolution while taking into account the size of the FoV, we calculated the number of PPD. A linear regression indicated that SS decreases for increasing PPD ($F(1,128)=7.455, p=0.007$). Visual inspection suggested that an exponential decay function of the form $\ln ({\text {SS}})=a\ + b\ \exp (-c\ \text {PPD})$ may account for the data better than a model of the form $\ln ({\text {SS}})=a+b\ \text {PPD}$. A comparison of the Akaike Information Criterion (AIC) values for these models supports this conclusion (336.392 and 356.312, respectively).

*Frame rate* Effects of FPS were tested in a similar manner as PPD. We compared the fit of a null model using only a constant as predictor to a linear and exponential decay model. There was no evidence for an effect of FPS ($F(1,110)=1.734, p=0.191$). Note however that there is very little data for high frame rates.

#### Mechanical fidelity

*Motion base* We can distinguish between static (fixed-base) simulators, dynamic (moving-base) simulators, and on-road conditions in real vehicles. Static simulators were used in 36 studies with a total of 94 experimental conditions, and average SS of 0.096 (Zou et al. 2021; Almallah et al. 2021; Sawada et al. 2020; Suwarno et al. 2019; Hohorst et al. 2019; Walch et al. 2017; Weidner et al. 2017; Romano et al. 2016; Bridgeman et al. 2014; Aykent et al. 2014; Park et al. 2005; Lin et al. 2002b; Colombet et al. 2016; Lucas et al. 2020; Sekar et al. 2020; Hartfiel and Stark 2019; Will et al. 2017; Schmieder et al. 2017; Eftekharifar et al. 2021; Jung et al. 2021; Kim et al. 2020; Kaufeld and Alexander 2019; Keshavarz and Hecht 2012; Moss and Muth 2011; Ujike and Watanabe 2011; Garcia et al. 2010; Kim and Park 2020; Stelling et al. 2020; Somrak et al. 2019; Mittelstaedt et al. 2018; Boustila et al. 2017; Emoto et al. 2008; Häkkinen et al. 2006; Damveld et al. 2010; Kovácsová et al. 2020; Klüver et al. 2016). Dynamic simulators were featured in 21 studies with a total of 43 conditions, and average SS of 0.077 (Talsma et al. 2022; Gemonet et al. 2021; Sawada et al. 2020; Jurisch et al. 2020; Parduzi et al. 2020; Hohorst et al. 2019; Romano et al. 2016; Aykent et al. 2014; Lucas et al. 2020; Sekar et al. 2020; Parduzi et al. 2019; Hartfiel and Stark 2019; Will et al. 2017; Jung et al. 2021; Kim et al. 2020; Kaufeld and Alexander 2019; Garcia et al. 2010; Stelling et al. 2020; Damveld et al. 2010; Kovácsová et al. 2020; Klüver et al. 2016). On-road conditions were present in 6 studies with a total of 11 conditions and average SS of 0.058 (Talsma et al. 2022; Zou et al. 2021; Gemonet et al. 2021; Benz et al. 2019; Stelling et al. 2020; Klüver et al. 2016). Statistical tests indicated that the difference in SS score between groups was not significant ($F(2,145)=2.286, p=0.105$).

Instead of classifying simulators as either static or dynamic, subgroups can also be made according to the number of DoF in which a simulator can provide motion. For a static simulator, the DoF are 0; dynamic simulators are subsequently ordered to the reported DoF. ANOVA indicated that there are no differences in SS depending on DoF ($F(5,131)=0.935, p= 0.461$)

#### Peripheral factors

*Physical fidelity* The physical fidelity of a simulator refers to its physical appearance. This contributes to simulator fidelity as an instrumented cabin is closer to a real vehicle than for example a desktop simulator with a monitor and steering wheel. We distinguish between simulators featuring a desktop setup (*n*=7, 18 conditions; mean SS = 0.1008) (Suwarno et al. 2019; Bridgeman et al. 2014; Park et al. 2005; Eftekharifar et al. 2021; Jung et al. 2021; Kim and Park 2020; Häkkinen et al. 2006), a vehicle mockup (*n*=17; 54 conditions; mean SS = 0.073) (Talsma et al. 2022; Zou et al. 2021; Almallah et al. 2021; Sawada et al. 2020; Parduzi et al. 2020; Hohorst et al. 2019; Walch et al. 2017; Weidner et al. 2017; Romano et al. 2016; Colombet et al. 2016; Lucas et al. 2020; Hartfiel and Stark 2019; Will et al. 2017; Stelling et al. 2020; Mittelstaedt et al. 2018; Damveld et al. 2010; Klüver et al. 2016), a complete cabin (*n*=10, 32 conditions; 0.099) (Gemonet et al. 2021; Jurisch et al. 2020; Aykent et al. 2014; Park et al. 2005; Lin et al. 2002b; Sekar et al. 2020; Parduzi et al. 2019; Schmieder et al. 2017; Garcia et al. 2010; Kovácsová et al. 2020), and actual vehicles (*n*=6, 15 conditions; mean SS = 0.074) (Talsma et al. 2022; Zou et al. 2021; Gemonet et al. 2021; Benz et al. 2019; Stelling et al. 2020; Klüver et al. 2016), and include a category ‘other’ for studies with participants in other positions or setups (*n*=8, 29 conditions; mean SS = 0.1029) (Kim et al. 2020; Kaufeld and Alexander 2019; Keshavarz and Hecht 2012; Moss and Muth 2011; Ujike and Watanabe 2011; Somrak et al. 2019; Boustila et al. 2017; Emoto et al. 2008), for instance lying in supine position (Emoto et al. 2008). Differences between the means in these conditions were not statistically significant ($F(4,143)=0.410, p=0.802$).

*Active vs. passive control* Rolnick and Lubow (1991) showed that participants who were exposed to rotational stimuli were less prone to develop motion sickness when they had control over the stimulation than when they did not have such control, suggesting that active control mitigates motion sickness. We therefore assess if SS differs depending on whether participants have a passive or active role in an experiment. Fifteen studies featured passive driving in 63 conditions, with average SS = 0.118 (Talsma et al. 2022; Zou et al. 2021; Sawada et al. 2020; Jurisch et al. 2020; Lin et al. 2002b; Colombet et al. 2016; Lucas et al. 2020; Eftekharifar et al. 2021; Jung et al. 2021; Kaufeld and Alexander 2019; Keshavarz and Hecht 2012; Ujike and Watanabe 2011; Stelling et al. 2020; Somrak et al. 2019; Emoto et al. 2008). Twenty-seven studies featured active driving in 85 conditions, with average SS = 0.073 (Gemonet et al. 2021; Almallah et al. 2021; Parduzi et al. 2020; Suwarno et al. 2019; Benz et al. 2019; Hohorst et al. 2019; Walch et al. 2017; Weidner et al. 2017; Romano et al. 2016; Bridgeman et al. 2014; Aykent et al. 2014; Park et al. 2005; Sekar et al. 2020; Parduzi et al. 2019; Hartfiel and Stark 2019; Will et al. 2017; Schmieder et al. 2017; Kim et al. 2020; Moss and Muth 2011; Garcia et al. 2010; Kim and Park 2020; Mittelstaedt et al. 2018; Boustila et al. 2017; Häkkinen et al. 2006; Damveld et al. 2010; Kovácsová et al. 2020; Klüver et al. 2016). Statistical test did not show a significant difference between these values ($F(1,146)=3.264, p=0.073$).

*Exposure duration* As motion sickness is a phenomenon that develops over time, it is possible that exposure duration, as a covariate, explains variability in SS scores. Contrary to this expectation, a model with exposure duration as single predictor yielded a *negative* coefficient ($-0.007$) for exposure duration measured in minutes. A possible explanation for a negative effect could be habituation (Reason and Brand 1975). However, a statistical test did not find this coefficient to be different from zero ($F(1,138)=2.119, p=0.148$).

*Age* It has been reported that younger individuals are more susceptible to motion sickness in a real vehicle (Kennedy and Lilienthal 1994; Schmidt et al. 2020; Paillard et al. 2013), whereas older individuals may be more susceptible to SS (Kennedy and Lilienthal 1994; Schmidt et al. 2020; Paillard et al. 2013). Although studies included in the present analyses did not provide sufficient detail to estimate effects of age at the level of the study, average age of the study sample is usually reported. We can therefore evaluate whether average age explains part of the variability in SS scores. Fits of a linear ($F(1,127)=3.393, p=0.068$) and non-linear ($F(1,126)=1.190, p=0.309$) exponential model of the form $\ln ({\text {SS}})=a\ + b\ \exp (-c\ \text {age})$ both yielded negative coefficients for the effect of age. However, these models did not perform better than a constant model including only an intercept.

### Meta-regression

#### Simulator sickness vs. overall fidelity

Ideally, the relation between multiple aspects of fidelity and SS would be assessed without making assumptions on the weight of any independent variable. One possible model for such an analysis, in Wilkinson notation Wilkinson and Rogers (1973), is:

1 $$\begin{aligned}&\ln ({\text {SS}}) \sim \text {base} * (\text {modality} + \text {FoV} + \text {stereoscopy} + \text {PPD}\nonumber \\& \quad + \text {FPS}) + (1 + ...\mid \text {study ID})\ . \end{aligned}$$

This includes as *fixed effects* a linear combination of effects for each aspect of visual fidelity, and allows these effects to differ depending on whether a simulator features a static or dynamic base. The last part of the equation, in brackets, reflects *random effects* that vary at the level of the study (study ID). These effects are at minimum a random intercept, and may include any study-dependent effects. For instance, the part between brackets could be a copy of preceding part of the equation following $\sim$. Successfully fitting this model requires sufficient observations for each possible combination of the levels of the (categorical) variables. In the following analysis, we exclude data from on-road conditions because there were only three observations from on-road conditions, with insufficient variation of fidelity aspects between them. In Table 1 we present the counts of the number of observations available for all combinations of the levels of each categorical aspect of fidelity. From the table, it is apparent that no observations were obtained for dynamic simulators that provide stereoscopic 3D cues by means of monitors or projection screens. This means that we cannot simultaneously include effects of visualization modality and stereoscopic cues on the one hand, and type of motion base on the other. Also, for two conditions only a single observation has been obtained (base = dynamic, stereoscopic = 0, modality = HMD; base = static, stereoscopic = 1, modality = monitor). This means that (co-)variances cannot be estimated.

**Table 1:** Breakdown of number of studies per type of simulator base; visualization modality; and whether or not visualizations were presented in stereoscopic 3D

Base	Stereoscopic	Modality	Conditions	References
Dynamic	0	Projection	12	Talsma et al. (2022), Jurisch et al. (2020), Aykent et al. (2014), Parduzi et al. (2019), Will et al. (2017), Damveld et al. (2010) and Klüver et al. (2016)
Dynamic	0	HMD	1	Stelling et al. (2020)
Dynamic	0	Monitor	15	Gemonet et al. (2021), Parduzi et al. (2020), Hohorst et al. (2019), Romano et al. (2016), Sekar et al. (2020), Parduzi et al. (2019), Garcia et al. (2010) and Stelling et al. (2020)
Dynamic	1	Projection	–	
Dynamic	1	HMD	13	Sawada et al. (2020), Parduzi et al. (2020), Lucas et al. (2020), Hartfiel and Stark (2019), Jung et al. (2021), Kim et al. (2020), Kaufeld and Alexander (2019), Stelling et al. (2020) and Kovácsová et al. (2020)
Dynamic	1	Monitor	–	
Static	0	Projection	19	Weidner et al. (2017), Aykent et al. (2014), Park et al. (2005), Will et al. (2017), Schmieder et al. (2017), Keshavarz and Hecht (2012), Boustila et al. (2017), Emoto et al. (2008), Damveld et al. (2010) and Klüver et al. (2016)
Static	0	HMD	5	Eftekharifar et al. (2021), Moss and Muth (2011) and Häkkinen et al. (2006)
Static	0	Monitor	19	Zou et al. (2021), Almallah et al. (2021), Suwarno et al. (2019), Hohorst et al. (2019), Walch et al. (2017), Romano et al. (2016), Bridgeman et al. (2014), Park et al. (2005), Sekar et al. (2020), Ujike and Watanabe (2011), Garcia et al. (2010), Kim and Park (2020), Somrak et al. (2019), Mittelstaedt et al. (2018), Häkkinen et al. (2006) and Klüver et al. (2016)
Static	1	Projection	17	Weidner et al. (2017), Lin et al. (2002b), Colombet et al. (2016), Schmieder et al. (2017), Keshavarz and Hecht (2012) and Boustila et al. (2017)
Static	1	HMD	30	Zou et al. (2021), Sawada et al. (2020), Suwarno et al. (2019), Walch et al. (2017), Weidner et al. (2017), Colombet et al. (2016), Lucas et al. (2020), Hartfiel and Stark (2019), Eftekharifar et al. (2021), Jung et al. (2021), Kim et al. (2020), Kaufeld and Alexander (2019), Kim and Park (2020), Stelling et al. (2020), Somrak et al. (2019), Mittelstaedt et al. (2018), Häkkinen et al. (2006) and Kovácsová et al. (2020)
Static	1	Monitor	1	Ujike and Watanabe (2011)

Note that not all conditions have been studied; we were unable to find studies where a dynamic simulator was used that featured stereoscopic 3D using a projection system or monitor

Similarly, to reliably estimate effects of the continuous independent variables, they should all be observed. This is complicated by the fact that not all studies provide information on the continuous predictors we wish to include: FoV, PPD and FPS. To deal with this, we calculate a visual fidelity score that combines the observed continuous predictors into one. We assume that fidelity increases with higher FoV, PPD and FPS. For FoV, the maximum value possibe is $360^\circ$; for PPD and FPS we set as maximum values the highest values that people may perceive under ideal conditions (128 and 500 Hz, respectively). We then scale the continuous predictors to the range between 0 and 1, using the maximum values for each predictor, and calculate an average for the available measures. Note that by calculating an average, we assume equal weighting for each of these factors. Also note that this approach is not feasible for the categorical variables because it is not apparent how they are ordered. Specifically, it is not known which of the different visualization modalities provides higher fidelity, and it is not clear how the presence or absence of stereoscopic cues should be weighted relative to the continuous cues.

Ultimately, sufficient information could be derived from the sample of studies to evaluate the following model:

2 $$\begin{aligned} \ln ({\text {SS}}) \sim \text {base}*(\text {modality} + \text {fidelity})\ + (1 \mid \text {study ID}). \end{aligned}$$

**Fig. 2:**
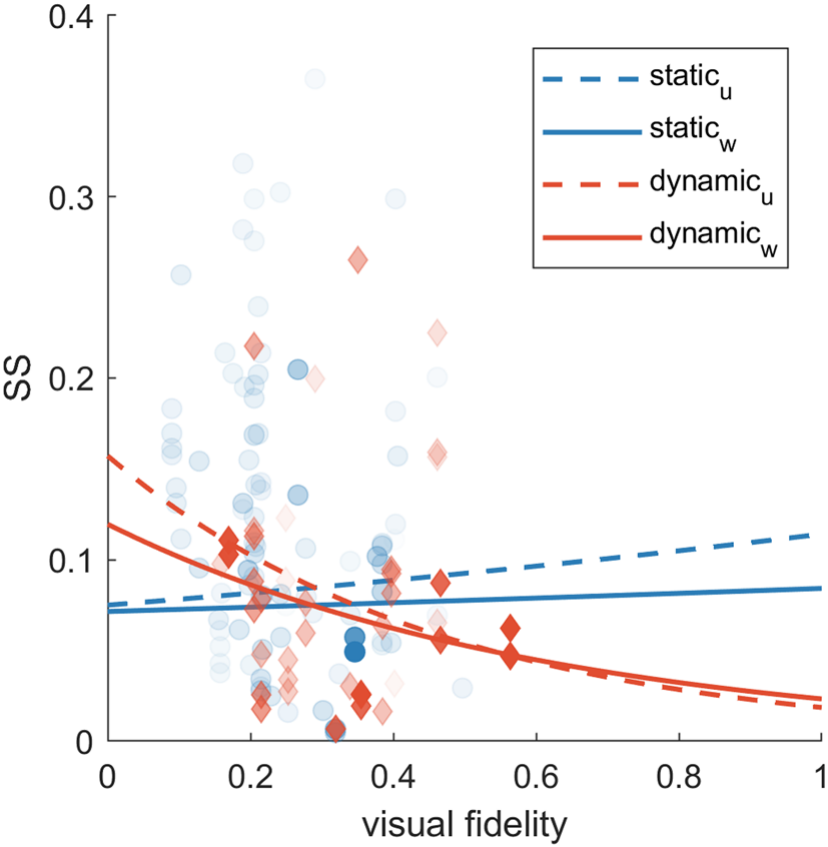
Visualization of the effects of visual fidelity, for static (blue) and dynamic (orange) motion bases. Dashed lines represent the unweighted model (Eq. 2); solid lines the weighted model. Model predictions were made on the $\ln ({\text {SS}})$ scale but converted back to the 0–1 range for visualization by taking the exponent. The transparency of the dots (i.e., individual observations) is proportional to the weight assigned to the observations in the meta-regression model, which is equal to the sample size of each study. For dynamic simulators, SS appears to decrease with increasing fidelity. This effect is not apparent for static simulators

Two versions of this model were fitted: an unweighted version, and a weighted version with weights assigned to each observation corresponding to the size of the sample from which it was derived, which is customary in meta-analyses (Schmidt and Hunter 2014; Borenstein et al. 2021). We distinguish between findings for the two versions by including a subscript *u* for the unweighted model, and a subscript *w* for the weighted model. For both versions of the model, fixed effect coefficients were similar. For the unweighted model, we found a negative significant effect for the fidelity score ($\beta _u=-2.136;\, F(1,129)=4.116, p=0.045$ ). This means that SS tends to *decrease* with increasing fidelity. An interaction effect was also observed between type of motion base and the fidelity score ($\beta _{u,\text {static}}=2.557;\, F(1,129)=4.218, p=0.042$). For the weighted model, the coefficient for visual fidelity was $\beta _w=-1.636$ ($F(1,129)=3.764, p=0.055)$ and the coefficient for the interaction between motion base and visual fidelity was $\beta _{w,\text {static}}=1.800$ ($F(1,129)=2.393, p=0.124$). For the weighted model, these coefficients were not statistically significant. Note that when the raw data from studies using an ME based method (i.e., data were not scaled according to the observed relation between FMS and SSQ scores) were used in this analysis, the effects were also significant for the weighted model. Although the disagreement between the models implies that more data is necessary, and caution should be exercised in drawing definitive conclusions, the interaction effect found in the unweighted model is in agreement with the hypothesized effect, and could nuance the effect of fidelity score; it suggests that the negative coefficient for fidelity score might effectively become zero for static simulators, and thus that increasing fidelity would have no effect on SS in static simulators, but can reduce sickness in dynamic simulators. The effects of visual fidelity and motion base are visualized in Fig. 2.

For the weighted version of the model, we did find a significant interaction effect between the type of motion base and visualization modality ($F(2,129)=3.412, p =0.036$). Although inspection of the coefficients themselves did not show significant differences, the value of the coefficient for monitors in static simulators, $\beta _{w, \text {static}, \text {monitor}} = -0.229$ implies that static desktop simulators produce less sickness, while static HMD simulators produce more sickness $\beta _{w, \text {static}, \text {HMD}} = 0.388$.

The present analysis covered studies performed over the last two decades. Given that simulator technology steadily improved during this time, it may be hypothesized that there exists a relation between publication year and fidelity. We explored this, but found no clear trend ($\rho =0.118, p=0.153$). Therefore, we believe it is better to focus analyses of fidelity on the actual technical specifications of a simulator.

#### Simulator sickness vs. visual modality and stereoscopy

**Fig. 3:**
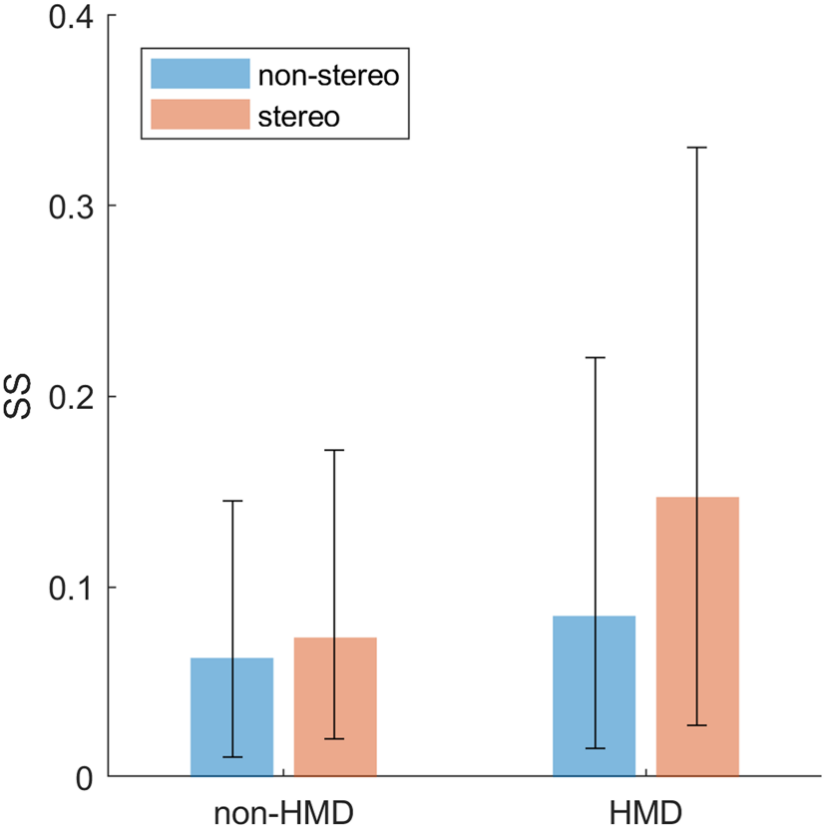
Marginal mean SS scores ($\pm \text {SD}$) of a model including visualization modality as being either an HMD or not, and whether or not visual cues were presented in stereoscopic 3D as dichotomous factors. Here we do not distinguish between the type of motion base. The statistical effects of these variables that were observed in individual analyses were not present in the joint analysis, although trends are consistent with linearly additive effects of these two variables

In the analyses of individual factors, it was observed that HMDs were more sickening than alternative visualization modalities, and also that visualizations presented in stereoscopic 3D were more sickening than visualization not presented in stereo. Because the majority of observations on 3D stereovision were obtained using HMDs (43/61), these variables may be confounding factors in analyses of either of them in isolation. In an attempt to disambiguate the role of stereoscopic 3D cues and HMDs versus monitors and projection screens, we fitted the following model:

3 $$\begin{aligned} \ln ({\text {SS}}) & \sim \text {HMD} * \text {stereoscopic} \\ &\quad + (1 + \text {HMD} * \text {stereoscopic} \mid \text {study ID}) , \end{aligned}$$

where both ‘HMD’ and ‘stereoscopic’ are dichotomous variables being true or false. The marginal means are presented in Fig. 3. The trends are consistent with linearly additive effects of stereoscopic 3D and HMDs, and show that stereoscopic stimuli tend to be more sickening, and also that HMDs are more sickening than alternative visualization modalities. However, ANOVA failed to show significant effects of either predictor variable, nor an interaction.

## Discussion

In the work presented here, we set out to provide a quantitative synthesis of effects of simulator characteristics on SS. The literature was searched for studies that varied aspects of visual fidelity for simulators with static or dynamic motion bases, and a sample of 41 studies was obtained from which data could be extracted. The analyses that were performed on this data allowed us to evaluate whether the available evidence supports the notion that high-fidelity visualizations cause more SS. In general, the data shows the opposite, namely that SS *reduces* with increased fidelity. Moreover, trends appear to support our hypothesis that this view may need to be further nuanced, and that the role of fidelity is different depending on whether a simulator features a static or dynamic base. However, even after synthesis of the data from many studies, the strength of the evidence was found to be limited, as the significance of findings depended on how data from different scales was combined and on the specific parametrization of the model (i.e., weighting observations or not). In the following, we first discuss the observations made in the exploratory analysis, followed by an evaluation of availability of data and the hypotheses. Finally, we make recommendations for future research.

### Exploratory analysis

To assess how simulator fidelity affect SS, we distinguished between dimensions of visual fidelity, mechanical fidelity, and a number of peripheral factors. For each of these dimensions, a selection of aspects was made that allow placement of simulators/studies along these dimensions. In the exploratory analysis, we assessed how SS varies with each of these factors, *not* simultaneously taking into account other variables.

In relation to visual fidelity, we found that HMDs produced more SS than alternative visualization modalities, and that visual stimuli presented in stereoscopic 3D were more sickening than non-3D stimuli. There was also evidence that more PPD lead to less sickness. FoV and FPS, by themselves, did not appear to affect the level of SS that was observed in studies.

A comparison between static and dynamic simulators to assess the role of mechanical fidelity per se also did not show any fundamental differences in the level of SS that would be attained in a study, and this result does not appear to change when considering the DoF featured by a simulator motion base. As the availability of more DoF may allow for closer approximation of a target trajectory, this evaluation thus does not provide support for the notion that “no motion is better than bad motion” (Spenny and Liebst 2003; Bos et al. 2008; Pais et al. 2009) when considering SS.

Similarly, no general effects were found for peripheral aspects, such as the physical fidelity (layout) of a simulator; whether participants had an active or passive role; the duration of exposure; or the average age of the participant sample.

Apart from the observed effects of some aspects of visual fidelity, which will be discussed in more detail below, the absence of general effects of other factors is not necessarily surprising. This is because researchers typically aim to control the level of sickness in studies; either because it is considered a nuisance in the study of other dependent variables, or to achieve a certain average target level of sickness, on top of which differential aspects of independent variables may be identified. To achieve a target level of sickness, researchers may tune the intensity of their motion paradigm and exclude from further analysis participants who either do not show any sickness or exceed a threshold level of sickness. In other words, some factors that may be expected to affect SS, such as exposure duration, may not show effects in a meta-analysis due to covariation of other variables.

The aspects considered here were chosen based on earlier work (Lin et al. 2007), with some modifications that were implemented with the aim to better reflect the nature of human perception. It should be noted that this list of included aspects is not exhaustive, and other aspects may certainly be relevant as well. In this regard, examples of potentially important aspects are the frequency content of motion stimuli (Irmak et al. 2021) and stimulation of non-visual and non-mechanical sensory modalities, notably audition. However, any meta-analysis is necessarily limited in scope to variables for which empirical work already exists, and that can be extracted from previous reports in practice.

### Meta-regression

An inventory of the studies yielded by the search revealed that observations are lacking or scarce for certain conditions (see Table 1). In particular, we were unable to find studies that used either projection systems or monitors to present visualizations in stereoscopic 3D, in dynamic motion simulators. Because of this, it was not possible to completely disentangle the effects of visualization modality, stereoscopy, and type of simulator base on SS. As a workaround, we now split this analysis in two; with a separate assessment of interactions between simulator base type, visualization modality, and visual fidelity on the one hand (Simulator sickness vs. overall fidelity), and an assessment of interactions between visualization modality and stereoscopy on the other (Simulator sickness vs. visual modality and stereoscopy). The former analysis provided evidence that the effect of visualization modality on SS differed depending on the type of motion base, with static simulators that use monitors provoking the least sickness. While the latter analysis does not allow definitive conclusions, it did suggest that effects of stereoscopy and type of visualization modality are additive. More precisely, visualizations presented in stereoscopic 3D via an HMD appear to be most sickening. Given the evidence for differential effects of visualization modality between motion base types, as well as for unequal effects of visual fidelity between motion bases, it is likely that the contrast between the effects of stereoscopy in HMDs and other visualization modalities is even stronger for static than for dynamic simulators. However, given the lack of data for these specific conditions, this cannot be ascertained.

Vis-à-vis our hypothesis, the unweighted meta-regression model indicated that SS decreases with increasing visual fidelity in dynamic simulators, but not necessarily in static simulators. This observation is consistent with the sensory conflict theory of motion sickness, as an increase in visual fidelity relative to absent mechanical motion also implies an increase in sensory conflict, whereas this is not necessarily the case when mechanical motion is also provided. A caveat here is that when mechanical motion cues are inconsistent with the visual motion cues (as they would be expected based on previous experience), then conflict can actually be increased.

Despite the consistency of these findings with conflict theory, it must be noted that, this effect was not significant in the weighted version of the model. A visualization of the data vs. the model fits suggests that in order to disambiguate the interactive effects of simulator base, data should be collected for either very low or very high visual fidelities (see Fig. 2). Here, the differential effect of motion base on the effect that visual fidelity has on SS is most apparent, whereas reliable data is scarce. It may be problematic to collect more data for very low fidelity visualizations, in particular for static simulators, because degraded visualizations as sole stimulus may not be sufficiently convincing to count as a simulation. However, more observations on SS could be obtained for high-fidelity visualizations and contrasted between static and dynamic simulators. One way to achieve this would be to mount a $360^\circ$ high-resolution and high-FPS camera to the roof of a vehicle, and to subsequently assess how motion sickness develops in the real vehicle, as well as in simulator renditions of the same drive in static and dynamic simulators, where the recorded videos are played back. Here it would also be of interest to use a stereoscopic camera system and to use monitors and/or projection systems capable of displaying stereoscopic 3D visualizations, to address the gap in the literature pointed out in the previous paragraph.

It is worthwhile noting that the observed differential effects of type of simulator base were robust to exclusion of the available data points for relatively high fidelity visualizations in dynamic simulators. These observations (approximate fidelity score of 0.6 in Fig. 2) were obtained from a study with a large sample, which was attributed considerable weight in the analysis (Parduzi et al. 2019).

### Conclusion

Overall, the present analyses indicate that higher fidelity simulators provoke less sickness. The comparisons between static and dynamic bases furthermore hint that a nuance may be required, namely that this conclusion could be primarily true for dynamic motion simulators, but not so much for static simulators. This finding is consistent with the sensory conflict theory of motion sickness. Apart from the considerations presented in the preceding discussion, observations made during the process of this analysis invite a number of recommendations for future research. These recommendations are not limited to research on the role of fidelity, but may generalize to research on motion sickness in general:

- it is common practice to exclude data from participants who aborted experiments from analyses. Information on the number of participants who aborted experiments along with their reasons for doing so and the level of sickness at the time of abortion could be useful to evaluate whether findings on SS are affected by censorship of such data;
- the calculation of sickness metrics was found to differ from original definitions in various studies. Although in some cases variations of calculations were intentional, for instance to achieve a better resolution for particular sickness symptoms, in other cases the variations appear to have been mistakes. This typically does not appear to invalidate results, as the calculations were performed consistently. In order to avoid confusion, it is recommended to report the exact equations used in the calculation of reported metrics.
- information on specific characteristics of equipment can usually be obtained from datasheets that are available from the OEM. Hence, by reporting the brand and model for each piece of equipment that is used in an experiment, other researchers may be able to retrieve information that may be irrelevant in an original study, but that may become relevant in future evaluations of studies with different research questions;
- Finally, we recommend to make experimental data along with any anonymized idiosyncratic demographic variables available to the scientific community. Making data available on the level of individual participants or observations allows other researchers to extract the maximum amount of information. Means and standard deviations are summaries of data that do not convey the number of observations nor how data are distributed. Consequently, any synthesis of individual data will be more powerful than a synthesis of summarized data. Various public repositories exist, and journals regularly offer options to include data in the form as online supplementary material to articles. A global registry of research data repositories is for example made available by the ‘re3data.org’ initiative (Pampel et al. 2013), and a list of various repositories is also made available in the Open Access Directory (OAD Simmons College (2022)). Example repositories are 4TU.ResearchData (Delft University of Technology Library 2022) and Data Archiving and Networked Services (DANS Koninklijke Nederlandse Akademie van Wetenschappen (2022)).

## Data Availability

A spreadsheet file including the search results, notes, and data used for the meta-regression is available as supplementary material.

## Footnotes

1 Simulator Sickness (SS) is a typical observation for static simulator environments, where only visual motion is presented. It is therefore sometimes referred to as Visually Induced Motion Sickness (VIMS). However, VIMS is not necessarily unique to simulators, and may also occur in, for example, a cinema. Because dynamic motion simulators can also provoke sickness through mechanical motion, we here prefer to use the term SS.
